# Lessons from a recent multicountry iatrogenic botulism outbreak

**DOI:** 10.2807/1560-7917.ES.2023.28.23.2300280

**Published:** 2023-06-08

**Authors:** Fabrizio Anniballi

**Affiliations:** 1 Istituto Superiore di Sanità, Department of Food Safety, Nutrition and Veterinary Public Health, National Reference Centre for Botulism, Rome, Italy

**Keywords:** botulism, iatrogenic botulism, botulinum toxin, outbreak

Way back in 1820, the German physician and poet Justinus Kerner was the first scientist to describe the gastrointestinal, autonomic and neuromuscular signs, symptoms and other clinical details of botulism in his monograph “Neue Beobachtungen über die in Württemberg so häufig vorfallenden tödlichen Vergiftungen durch den Genuss geräucherter Würste” [New observations on the lethal poisoning frequently occurring in Württemberg through the consumption of smoked sausages]. Through observations and self-experiments, Kerner deduced the potential use of the ‘sausage poison’ (now botulinum neurotoxin, BoNT) for treating hyperactivity and hyperexcitability of the motor and autonomic nervous system [1]. However, therapeutic use of the BoNT would have to wait until 1989, when the United States’ Food and Drug Administration (FDA) approved it for the treatment of blepharospasm and strabismus, based on the experiments published by the ophthalmologist Alan B. Scott in 1973 [2]. From 1973 to date, scientific research on the therapeutic use of BoNTs has increased massively. Indeed, a search on “botulinum toxin therapeutic” in the PubMed database on 29 May 2023, retrieved about 13,000 published papers.

Nowadays, at least eight different commercial formulations of BoNT are available worldwide [3]. Although these formulations have been approved for a limited number of clinical applications, their off-label and experimental use involve more than fifty conditions in the areas of ophthalmology, neurology, plastic surgery and dermatology, orthopaedics, gastroenterology, urology and gynaecology, and rheumatology [3]. The success of BoNT as therapeutic lies in the fact that it is highly selective for nerve endings, powerfully inhibits neurotransmitter release, diffuses from the site of injection only to a limited extent and produces a reversible effect [4,5]. Generally, the first effects (both positive and adverse) of BoNT treatment can be observed within 24–72 h after its injection, even if, in some cases, they can be delayed for up to 2 weeks, whereas the peak effect is reached before 2–4 weeks. Positive effects commonly persist for 3–6 months but depend on the type of toxin used, the dose, and the mode of administration [5].

BoNT treatment is generally well-tolerated by patients; however, some side effects have been described. The most common ones, such as erythema, oedema, bruising and pain, are usually localised on the side of injection and resolve without treatment within a few days [3]. On rare occasions, however, the toxin can diffuse around the site of injection reach lymphatic circulation and lead to systemic adverse effects such as muscle weakness, allergic reactions, and typical symptoms of botulism (i.e. diplopia, bilateral ptosis, slow reactive pupils, accommodation deficit, dysphonia, dysphagia, dry mouth, generalised weakness) [6,7]. Systemic side effects producing symptoms of botulism have been described rarely and were usually associated with injecting unlicensed drug preparations or errors in administering the therapeutics (i.e. shortened inter-dose interval, a larger volume of fluid injected, higher single injection doses) [6,8-10]. Recently, a large travel-associated European outbreak of iatrogenic botulism occurred in patients treated with BoNT for weight reduction in Türkiye [11]. As reported by the European Centre for Disease Prevention and Control (ECDC) and the World Health Organization Regional Office for Europe (https://www.ecdc.europa.eu/en/news-events/botulism-iatrogenic-update-cases-europe-march-2023), 87 cases have been reported by Germany, Austria, France, Switzerland and Türkiye. The article by Dorner et al. in this issue describes the investigations of 34 cases outside Türkiye [11].

Available information on this large and unusual outbreak allows us some considerations and reiterates some concepts: although rare, botulism is a life-threatening disease. Physicians who suspect botulism on clinical grounds should promptly collect clinical samples suitable for laboratory confirmation and notify the suspected case to health authorities, allowing well-timed epidemiological investigations. Timely anamnesis and epidemiological investigation increase the probability of limiting or even better, avoiding the spread of the disease because the patient’s recollection may still be precise, and dysarthria or respiratory failure has not yet appeared [12]. At this time, health authorities can successfully launch campaigns through traditional and social media to inform the public and actively find new cases early. In addition, a prompt formulation of the clinical suspicion allows physicians to obtain laboratory confirmation at an early stage of the disease, which enables adequate disease management and helps to avoid pointless treatments. An accurate medical history and epidemiological investigations also enable the rapid activation of national and international early warning systems, as occurred during the iatrogenic botulism outbreak in Türkiye.

Iatrogenic botulism is an underestimated and under-reported disease for several reasons. In some countries, notification of this botulism form is not mandatory, and physicians, especially those responsible for BoNT administration, may not report cases on their own initiative. In some countries, iatrogenic botulism is notified only to the national medicines agencies as adverse drug reaction. For example, from June 2022 to 29 May 2023, the European database of suspected adverse drug reactions (https://www.adrreports.eu/en/) reported 5,343 cases in the European Union/European Economic Area, while only two papers on iatrogenic botulism appeared in PubMed [13,14]. Although the most common side effects recorded after BoNT administration are mild and cannot be considered iatrogenic botulism, the difference in the aforementioned numbers is incommensurable, supporting the hypothesis of under-reporting.

Close collaboration and cooperation among the different health authorities at national and international levels in sharing available information are essential for the rapid reconstruction and management of outbreaks both at national and international levels. Although iatrogenic botulism is not infectious, health authorities in the recent outbreak successfully used the ECDC EpiPulse portal to disseminate information and monitor the event.

Laboratory diagnosis of iatrogenic botulism is particularly challenging because of the low amounts of circulating toxin; however, the German National Consultant Laboratory for neurotoxin-producing clostridia at the Robert Koch Institute successfully detected BoNT in the serum of some suspected cases. Indeed, this laboratory rapidly optimised an alternative method to the mouse bioassay based on Endopep-mass spectrometry suitable for confirmation of iatrogenic botulism. This important achievement could be useful for all laboratories involved in botulism confirmation, overcoming the drawbacks encountered in those laboratories that rely solely on the use mouse bioassay for diagnostic purposes.

Obesity is a worldwide relevant public health concern; since lifestyle modification is maybe ineffective in reducing morbidity, the most suitable treatment in such cases remains bariatric surgery, albeit being invasive [15]. In searching for non-invasive or less invasive treatments for obesity, intragastric administration of BoNT has been evaluated. The principle of this treatment lies in the capability of BoNT to paralyse the stomach muscles, causing the reduction of gastric emptying and improving satiety [15]. Although the BoNT stomach injection for body weight loss is a clinical practice that started about 20 years ago, it is still an off-label treatment. Several studies, including clinical trials, have been published on this issue; however, the available data regarding the efficacy of this treatment are conflicting [15-20].

Regarding the administration of pharmaceuticals in an off-label fashion, the relevant European Union legislation does not regulate how medicinal products are used in medical practice. Prescribing a medicinal product, on-label or off-label, is a decision taken within the relationship between the healthcare professional and the patient [21]. Healthcare professionals should provide all the details of the treatment they intend to prescribe or carry out and give the patients the necessary elements to make an informed decision. Medical societies and healthcare professionals should develop and disseminate informative material such as posters, videos, brochures and divulgative publications to increase awareness of iatrogenic botulism among the general population.

To increase patient safety, care equity, standardise clinical practices and improve BoNT therapy, development and updating of consensus guidelines and their dissemination should be promoted to enable early detection and investigation of a condition that is relevant both for the individual as well as public health.

## References

[1] Erbguth FJ. From poison to remedy: the chequered history of botulinum toxin. J Neural Transm (Vienna). 2008;115(4):559-65. 10.1007/s00702-007-0728-2 17458494

[2] Scott AB Rosenbaum A Collins CC. Pharmacologic weakening of extraocular muscles. Invest Ophthalmol. 1973;12(12):924-7. 4203467

[3] Bach K Simman R. The Multispecialty Toxin: A Literature Review of Botulinum Toxin. Plast Reconstr Surg Glob Open. 2022;10(4):e4228. 10.1097/GOX.0000000000004228 35402123 PMC8987218

[4] Dressler D. Clinical applications of botulinum toxin. Curr Opin Microbiol. 2012;15(3):325-36. 10.1016/j.mib.2012.05.012 22770659

[5] Pirazzini M Rossetto O Eleopra R Montecucco C. Botulinum Neurotoxins: Biology, Pharmacology, and Toxicology. Pharmacol Rev. 2017;69(2):200-35. 10.1124/pr.116.012658 28356439 PMC5394922

[6] Leonardi L Haggiag S Petrucci A Lispi L. Electrophysiological abnormalities in iatrogenic botulism: Two case reports and review of the literature. J Clin Neurosci. 2019;60:138-41. 10.1016/j.jocn.2018.10.059 30348587

[7] Palazón-García R Benavente-Valdepeñas AM. Botulinum Toxin: From Poison to Possible Treatment for Spasticity in Spinal Cord Injury. Int J Mol Sci. 2021;22(9):4886. 10.3390/ijms22094886 34063051 PMC8125452

[8] Chertow DS Tan ET Maslanka SE Schulte J Bresnitz EA Weisman RS Botulism in 4 adults following cosmetic injections with an unlicensed, highly concentrated botulinum preparation. JAMA. 2006;296(20):2476-9. 10.1001/jama.296.20.2476 17119144

[9] Szuch E Caress JB Paudyal B Brashear A Cartwright MS Strowd RE 3rd. Head drop after botox: Electrodiagnostic evaluation of iatrogenic botulinum toxicity. Clin Neurol Neurosurg. 2017;156:1-3. 10.1016/j.clineuro.2017.02.012 28273554 PMC10335596

[10] Rashid EAMA El-Mahdy NM Kharoub HS Gouda AS ElNabarawy NA Mégarbane B. Iatrogenic Botulism Outbreak in Egypt due to a Counterfeit Botulinum Toxin A Preparation - A Descriptive Series of Patient Features and Outcome. Basic Clin Pharmacol Toxicol. 2018;123(5):622-7. 10.1111/bcpt.13048 29786953

[11] Dorner MB Wilking H Skiba M Wilk L Steinberg M Worbs S A large travel-associated outbreak of iatrogenic botulism in four European countries following intragastric botulinum neurotoxin injections for weight reduction, Türkiye, February to March 2023. Euro Surveill. 2023;28(23):2300203. 10.2807/1560-7917.ES.2023.28.23.2300203 PMC10318948 37289431

[12] Scalfaro C Auricchio B De Medici D Anniballi F. Foodborne botulism: an evolving public health challenge. Infect Dis (Lond). 2018;1-5. 30499356 10.1080/23744235.2018.1524584

[13] Mohapatra RK Mishra S Tuglo LS. Surge in iatrogenic botulism cases in Europe: Threat perceptions and salient countering measures. New Microbes New Infect. 2023;53:101142. 10.1016/j.nmni.2023.101142 37216025 PMC10195973

[14] Gonul Oner O Gudek HC Erturk Cetin O Demir S. Iatrogenic Botulism: A Case Treated With Botulinum Antitoxin. Clin Neuropharmacol. 2023;46(2):82-4. 10.1097/WNF.0000000000000535 36728838

[15] Sánchez-Torralvo FJ Vázquez-Pedreño L Gonzalo-Marín M Tapia MJ Lima F García-Fuentes E Endoscopic Intragastric Injection of Botulinum Toxin A in Obese Patients Accelerates Weight Loss after Bariatric Surgery: Follow-Up of a Randomised Controlled Trial (IntraTox Study). J Clin Med. 2022;11(8):2126. 10.3390/jcm11082126 35456220 PMC9027998

[16] Vargas EJ Bazerbachi F Calderon G Prokop LJ Gomez V Murad MH Changes in Time of Gastric Emptying After Surgical and Endoscopic Bariatrics and Weight Loss: A Systematic Review and Meta-Analysis. Clin Gastroenterol Hepatol. 2020;18(1):57-68.e5. 10.1016/j.cgh.2019.03.047 30954712 PMC6776718

[17] Gui D De Gaetano A Spada PL Viggiano A Cassetta E Albanese A. Botulinum toxin injected in the gastric wall reduces body weight and food intake in rats. Aliment Pharmacol Ther. 2000;14(6):829-34. 10.1046/j.1365-2036.2000.00765.x 10848669

[18] Topazian M Camilleri M Enders FT Clain JE Gleeson FC Levy MJ Gastric antral injections of botulinum toxin delay gastric emptying but do not reduce body weight. Clin Gastroenterol Hepatol. 2013;11(2):145-50.e1. 10.1016/j.cgh.2012.09.029 23063681 PMC3552074

[19] Bang CS, Baik GH, Shin IS, Kim JB, Suk KT, Yoon JH, et al. Effect of intragastric injection of botulinum toxin A for the treatment of obesity: a meta-analysis and meta-regression. Gastrointest Endosc. 2015;81(5):1141-9.e1-7. 10.1016/j.gie.2014.12.025 25765772

[20] Bang CS Baik GH Shin IS Kim JB Suk KT Yoon JH Effect of intragastric injection of botulinum toxin A for the treatment of obesity: a meta-analysis and meta-regression. Gastrointest Endosc. 2015;81(5):1141-9.e1, 7. 10.1016/j.gie.2014.12.025 25765772

[21] European Commission. Directorate-General for Health and Food Safety. STAMP Commission Expert Group. Off-label use of medicinal products. Brussels: European Commission. 14 Mar 2017. Available from: https://health.ec.europa.eu/system/files/2017-04/stamp6_off_label_use_background_0.pdf

